# Effects of frequent assessments on the severity of suicidal thoughts: an ecological momentary assessment study

**DOI:** 10.3389/fpubh.2024.1358604

**Published:** 2024-05-17

**Authors:** Tengwei Chen, Lu Niu, Jiaxin Zhu, Xiaofei Hou, Haojuan Tao, Yarong Ma, Vincent Silenzio, Kangguang Lin, Liang Zhou

**Affiliations:** ^1^Department of Social Medicine and Health Management, Xiangya School of Public Health, Central South University, Changsha, China; ^2^Tianjin Anding Hospital, Mental Health Center of Tianjin Medical University, Tianjin, China; ^3^National Clinical Research Center for Mental Disorders, and Department of Psychiatry, The Second Xiangya Hospital of Central South University, Changsha, China; ^4^The Affiliated Brain Hospital of Guangzhou Medical University, Guangzhou, China; ^5^Urban-Global Public Health, Rutgers School of Public Health, Rutgers, The State University of New Jersey, Newark, NJ, United States; ^6^School of Health and Life Sciences University of Health and Rehabilitation Sciences, Qingdao, China

**Keywords:** suicidal thought, ecologic momentary assessment, intensive repeated measurement, Bayesian multilevel model, suicide

## Abstract

**Objective:**

In recent years, there has been a significant increase in research using ecological momentary assessment (EMA) to explore suicidal thoughts and behaviors (STBs). Meanwhile, concerns have been raised regarding the potential impacts of frequent and intense STBs assessments on the study participants.

**Methods:**

From November 2021 to June 2023, a total of 83 adolescent and young adult outpatients (M_age_ = 21.0, SD_age_ = 6.3, 71.1% female), who were diagnosed with mood disorders, were recruited from three psychiatric clinics in China. Smartphone-based EMA was used to measure suicidal thoughts three times per day at randomly selected times. We examined the change of suicidal thoughts in each measurement and within 1 day to evaluate potential adverse effects using Bayesian multilevel models.

**Results:**

The 3,105 effective surveys were nested in 83 participants (median follow-up days: 14 days). The results of two-level models indicated that suicidal thoughts decreased during the monitoring period. However, this effect varied among different individuals in the two-level model.

**Conclusion:**

Our findings did not support the notion that repeated assessment of suicidal thoughts is iatrogenic, but future research should continue to investigate the impact of frequent assessment on suicidal thoughts, taking into account individual differences and utilizing larger sample sizes.

## 1 Introduction

Suicidal thoughts and behaviors (STBs) are a major global health concern, leading to a significant loss of life each year (1). In response, there is a pressing need for enhanced research efforts to understand and prevent STBs effectively. New technologies, such as smartphone apps, offer innovative ways to study STBs in real time, shedding light on their dynamic nature (2).

With the growing use of ecological momentary assessments (EMA) on STBs, an important question arises regarding the impact of frequent assessments on individuals (3, 4). Previous research has provided promising findings, indicating that single-point inquiries about suicide are not inherently harmful or increase the risk of suicide (5–7). However, there is limited evidence on the effects of intensive questioning about suicidal thoughts over a short period.

Law et al. (8) conducted a randomized controlled trial to investigate the potential harmful effects of repeated assessments of suicidal ideation among individuals with borderline personality disorder. Similarly, Coppersmith et al. (9) innovatively studied the effects of frequent assessments of suicidal ideation on a high-resolution time scale. While both studies did not show any harm in repeatedly assessing suicidal thoughts, it is important to note that the existing evidence is based on English-speaking contexts. This limits the applicability of the findings to diverse cultures and languages. Specifically, while existing research indicates that frequent evaluations of suicidal ideation have not shown adverse effects on most youth (10, 11), studies involving adolescents are relatively scarce, and there is insufficient research evidence available (3). Additionally, it is crucial to acknowledge that the effects of frequent evaluation can vary among individuals (3).

Therefore, this study aimed to investigate the potential impact of intensive monitoring on the severity of suicidal thoughts in the short term. Specifically, we aimed to answer three questions: (a) How does the severity of suicidal thoughts change as the number of surveys during the monitoring period increases? (b) How does it change daily? (c) Are there individual differences in these levels of change?

## 2 Method

### 2.1 Participants

From November 2021 to June 2023, the study recruited outpatients with mood disorders from psychiatric clinics in three hospitals in Changsha, Guangzhou, and Tianjin, China. The inclusion criteria for participation in the study were: (1) aged 12 years old or above; (2) experienced suicidal thoughts within the past 2 weeks, (3) had a diagnosis of a mood disorder, and (4) possessed a smartphone (Android or iOS). Individuals were excluded if they had previous or current psychotic symptoms or other psychiatric illnesses, were currently experiencing a manic episode, were unable to provide informed consent or answer self-assessment questions due to cognitive impairment, or were determined by psychiatrists to be at high risk of suicide and in need of immediate intensive intervention or hospitalization.

During the study period, 89 patients carried out the ecological momentary assessment (EMA). 47 participants were monitored for 28 days (November 2021 to November 2022) and 42 participants were monitored for 14 days (February 2023 to June 2023). We summarized and analyzed the two sets of data in this study. Among the 89 participants, 6 patients were excluded from the analysis due to a response rate lower than 20%. Of the remaining 83 patients, 17 dropped out, with an average monitoring time of 11.24 days. The detailed reasons for dropping out see Supplementary Table S1.

### 2.2 Study procedure

#### 2.2.1 Baseline assessment

Before the study, we acquired the informed consent of the participants and additionally obtained the assent of the guardians of adolescent participants. Then we conducted baseline surveys collecting socio-demographics and suicide-related risk factors. Regarding clinical diagnoses, patients were examined and diagnosed by psychiatrists during their visits, based on the *International Statistical Classification of Diseases and Related Health Problems* (ICD-10) (12). Then, the investigator retrieves the diagnoses from their medical records. The Beck Scale for Suicide Ideation-Chinese Version (BSI-CV) was utilized to assess suicidal ideation within a week (13). Depression and anxiety were measured using Patient Health Questionnaire-9 (PHQ-9) (14) and Generalized Anxiety Disorder-7 (GAD-7) (15), respectively. The baseline characteristics of the study population are shown in Table 1.

**Table 1:** Baseline characteristics of the participants (*n* = 83).

Variables	M ± SD(*n*/%)
Age	21.1 ± 6.3
Gender	
Male	24 (28.9)
Female	59 (71.1)
Education	
Junior high school	15 (18.1)
Senior high school/technical secondary school/higher vocational school	29 (34.9)
Junior college/university	39 (47.0)
Diagnose	
Depressive disorder	58 (69.9)
Bipolar disorder	25 (30.1)
Suicidal ideation within a week (BSI-CV)^a^	15.2 ± 6.9
Depression (PHQ-9)^b^	19.3 ± 4.9
Anxiety (GAD-7)^c^	14.7 ± 5.2
Suicidal attempt	
Lifetime	44 (53.0)
In the last year	31 (37.3)

a The BSI-CV was rated on a scale of 0 to 38.

b The PHQ-9 was rated on a scale of 0 to 27.

c The GAD-7 was rated on a scale of 0 to 21.

#### 2.2.2 EMA design and measurement

Each participant registered on the Wechat mini program (Xunkang assessment system, details see Supplementary Figures S1, S2) and filled out questionnaires online. Each day, participants received 3 momentary surveys, which were completed at random intervals (within a 20-min window of receiving the prompt) during three periods the participants suggested they would be available (usually from 9:00 am to 9:00 pm). During the periods, three surveys occurred with a minimum interval of 60 min between prompts. The participants had 20 min to respond. If a participant did not complete the survey after the initial reminder, three additional reminders would be sent within 20 min. The reminders were delivered via a mini-program and APP with access to the survey. In addition, participants can self-initiate the survey to log STBs or NSSIs at the moment, when they had STBs or NSSI at any time other than the established sampling survey time. The surveys measured suicidal thoughts and related factors. Suicidal thought was measured by one item, which was adapted from the ninth item of the Patient Health Questionnaire (PHQ-9, 14) (i.e., Do you want to die or end your life in some way at this moment?). The item was measured from 0 (none) to 6 (very intense) scale. Positive and negative moods [PANAS, (16)] were measured on a 5-point scale. Self-harm behaviors were measured by dichotomy.

### 2.3 Incentive and ethical consideration

During the survey, participants who maintained a weekly response rate of 75% or higher would receive 100 Chinese Yuan (CNY). After the baseline assessment, researchers worked with each participant together for the *Patient Safety Plan* [SPI, (17)]. Our study has a risk and safety monitoring system. During the monitoring period, responses to all suicide-related EMA questions were reviewed three times daily during the monitoring period (from 9 a.m. to 9 p.m.). Based on the study of Glenn et al. (9), risk cutoffs for suicide-related EMA items were utilized to create a standardized method for monitoring and assessing risk. Any endorsement of suicidal thoughts over 4 (range 0–6) and self-harm behaviors (no matter with or without suicidal intent) was flagged as high-risk. If the response to these items were flagged for risk, the research team (PI and main investigators) would instantly receive the auto-message (including participants’ relevant information) and initiate the follow-up contact.

Investigators (trained MSc students in psychiatry or social medicine) promptly reached out to the participants via WeChat or phone within 15 min when receiving a warning text-message. Investigators assessed the participant’s status and provided guidance on utilizing their personalized safety plan. If there was no response from participants in 15 min, their parents were contacted. Additionally, based on their risk status (e.g., imminent risk), their parent would be also contacted.

According to the *Consensus Statement on Ethical & Safety Practices for conducting digital monitoring studies with people at risk of suicide and related behavior* and previous studies (18), if participants self-initiate a survey to log STBs or NSSIs at the moment during nighttime (ie. 9 pm-9 am), our team follow-up within 12 h. This timeframe was included in the content in detail, and participants and their parents were well-informed.

Following these contacts, the investigator timely reported the participants’ status to the professionals in our research team (including experts in suicide prevention, crisis intervention, and psychiatrists). Based on the participants’ condition, the expert team would provide recommendations, such as scheduling a follow-up appointment at the outpatient clinic or considering inpatient care.

Approvals were obtained from the Institutional Review Boards (IRBs) of the Xiangya School of Public Health, Central South University (XYGW-2021-73 and XYGW-2022-39), the Affiliated Brain Hospital of Guangzhou Medical University (2021–089) and Tianjin anding hospital (2023–01 and 2023–02). Written informed consent was obtained from all participants (and one of their parents if they were under 18 years old).

### 2.4 Statistical analysis

We employed Bayesian multilevel models to examine the research questions in this study. For question (a), we applied a two-level ordinal model (Model 1) that each time-based measurement was nested within individuals. For question (b), we extended our analysis to a three-level ordinal model (model 2), that the measurements were nested in daily surveys and individuals. For question (c), the observation number was treated as a random effect in both multilevel models to derive variations in the changes of suicidal thoughts among individuals.

Suicidal thoughts served as the outcome variable, and we explored its relationship with the number of surveys. Additionally, we included momentary positive and negative emotions, and suicidal thought at the previous moment, to examine their potential influence on the association between the survey number and suicidal thoughts.

In addition, given that the median participation duration was 14 days, we divided the 28-day survey into two equal intervals of 14 days each and reanalyzed the data.

This study utilized the No-U-Turn sampler (19) for model estimation through the brms (20) package and Stan (21), based on the algorithm of Markov chains Monte Carlo (MCMC). MCMC diagnostics and model examination, including effective sample size (ESS), Rhat (Supplementary Table S2), and posterior predictive check (Supplementary Figure S3), are crucial steps in Bayesian modeling (22), and their results and model parameter settings are presented in the Supplement. Statistical indexes of parameter posterior distribution, such as median and 95% highest intensity intervals (95% HDI, the true parameter has a 95% probability of being within this interval), were employed. The study used R (23) for statistical analysis and data visualization, brms (20) package for Bayesian hierarchical model analysis. The parameter was considered statistically significant if the 95% HDI of the posterior distribution did not include zero.

## 3 Results

### 3.1 Descriptive results of momentary surveys

**Figure 1:**
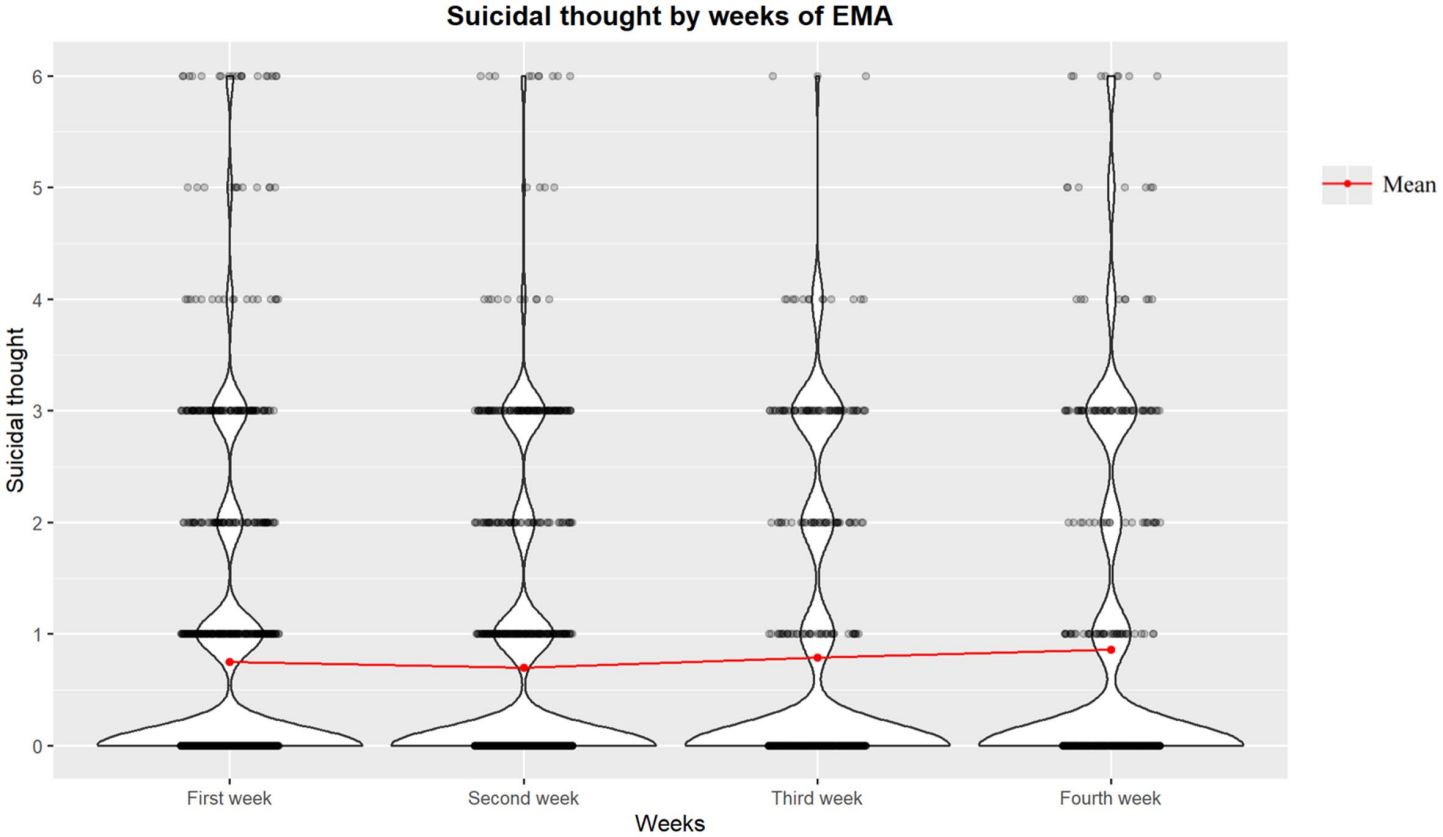
Weekly distribution of severity of suicidal thoughts.

The median duration of completed EMA days among participants was 14 days (range = 3–28 days). Specifically, they completed a total of 3,105 assessments (average 2.03 times per person per day), which comprised 67.6% of all the surveys that were prompted. The mean score for suicidal thoughts was 1.75 (SD = 1.29). In total, suicidal thoughts were reported in 806 surveys, an average of 9.7 times per person. 86.75% (72/83) of participants reported suicidal thoughts at least once. The ICC and RMSSD suicidal thoughts, respectively, are 0.53 and 0.84 (range: 0 to 3). As shown in Figure 1, the result of the Kruskal-Wallis test did not reveal any significant differences in the distribution of suicidal ideation severity over weeks (χ2 = 0.81, *p* = 0.85).

### 3.2 The effect of frequent assessments on suicidal thoughts

For question (a), we discovered a significant negative association between the number of surveys and the severity of suicidal thoughts [median *β* = −0.03, 95%HDI (−0.05, −0.01)], after controlling for variables such as momentary emotion and suicidal thought at t-1. This result indicated that as the number of surveys increased, there was a decrease in the severity of suicidal thoughts. For question (b), we did not find a similar association between the number of surveys and suicidal thoughts within a day [median *β* = −0.14, 95% HDI (−0.34, 0.06)]. The fixed effect results of the above models are presented in Table 2.

**Table 2:** Bayesian cumulative ratio model of the testing association between the observation number and suicidal thought.

	Model 1^c^		Model 2^c^	
Variable	Median	95%HDI	Median	95%HDI
Fixed effects				
(Intercept 1)	1.94	1.24, 2.67	2.46	1.69, 3.28
(Intercept 2)	4.21	3.48, 4.96	4.76	3.96, 5.64
(Intercept 3)	5.58	4.79, 6.33	6.12	5.24, 7.02
(Intercept 4)	8.69	7.83, 9.55	9.30	8.31, 10.37
(Intercept 5)	9.59	8.73, 10.55	10.22	9.15, 11.32
(Intercept 6)	10.34	9.38, 11.29	10.99	9.90, 12.18
Suicidal thought at t-1	0.46	0.33, 0.59	0.50	0.36, 0.65
Number of surveys	−0.03	−0.05, −0.01	−0.14	−0.34, 0.06
Positive emotion	−0.88	−1.17, −0.57	−0.78	−1.10, −0.48
Negative emotion	1.77	1.53, 2.02	1.87	1.59, 2.17
Random effects				
Level-2				
σ_intercept_	2.35	1.82, 2.91	0.41	0.00, 0.86
σ_observation number_	0.04	0.03, 0.06	0.14	0.00, 0.34
Level-3				
σ_intercept_			2.68	2.04, 3.37
σ_observation number_			0.13	0.00, 0.36
Loo-R^2^^a^	0.720		0.716	
Looic^b^	2733.3		2811.7	

a Loo-R^2^is the R^2^ adjusted by Leave-One-Out Cross-Validation (LOO-CV).

b Looic transforms the Bayesian LOO estimate of expected log pointwise predictive density into an information deviation scale.

c Since suicidal thoughts at the t-1 were included in the independent variable, the sample size was 83 participants with 2266 observations.

### 3.3 Individual heterogeneity

**Figure 2:**
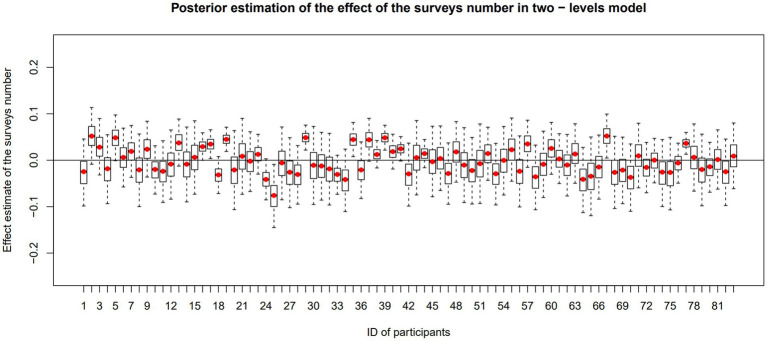
Effect of surveys number on suicidal thought severity in individuals. This chart is based on results of the two-level modal. In each box, red rot represents the mean value of the individual posterior distribution. The upper and lower boundaries of the box represent the 75th and 25th percentiles of the distribution, respectively. The dashed line has upper and lower ends which indicate the maximum and minimum values. If the box or the dashed line crosses the zero line, it indicates that the effect is not statistically significant, and vice versa.

For question (c), the two-level model revealed a statistically significant random effect (Table 2), indicating that the association between the severity of suicidal thoughts over time varied among individuals [median σ = 0.04, 95%HDI (0.03, 0.06), Figure 2]. In the three-level model, the result of the random effect did not reveal significant individual heterogeneity in the daily trajectories of suicidal thoughts [median *σ* = 0.14, 95%HDI (0.00, 0.34)].

### 3.4 Sensitive analysis

We examined the trajectories of suicidal thoughts during the first 14 days and the later 14 days. The results in Supplementary Table S3. In the two-level model, it indicated a negative association between survey number and the severity of suicidal thoughts in the first 14 days [median *β* = −0.04, 95%HDI (−0.08, −0.02)]. But it did not suggest significant correlations in the second 14 days [median *β* = −0.03, 95%HDI (−0.08, 0.02)]. In the three-level model, no statistically significant correlations were observed within a day [The first 14 days: median *β* = −0.17, 95%HDI (−0.44, 0.06); The second 14 days: median *β* = −0.21, 95%HDI (−0.67, 0.16)].

## 4 Discussion

This study shed light on the potential impact of intensive monitoring on the severity of suicidal thoughts in the short term, considering the long-standing concern that asking individuals about suicide might be harmful. Our findings did not support the notion that repeated assessment of suicidal thoughts is iatrogenic, which is consistent with previous research (8, 9). However, there are still several points that need to be considered when drawing the conclusion.

Our study found that there may be individual heterogeneity regarding the relationship between survey frequency and the severity of suicidal thoughts. Throughout the study, a small number of participants provided negative feedback, which was consistent with previous researches (10, 24). Four patients expressed emotional distress, with one reporting a heightened focus on their negative emotions and potentially amplifying them while filling out the questionnaire. Another patient felt uncomfortable when disclosing their thoughts and emotions during the EMA. Additionally, two patients felt a sense of being monitored and were concerned that others would become aware of this study (Zhu et al., manuscript submitted).^1^ These findings emphasize the importance of considering the impact of repeated assessments at an individual level. It can help inform the design of feasible, acceptable, and psychologically safe EMA protocols for suicide-related research. Future research should carefully consider participants’ distinct feedback to repeated assessments, including emotional, psychological, and behavioral aspects, and address any issues promptly.

According to *Consensus Statement on Ethical & Safety Practices for conducting digital monitoring studies with people at risk of suicide and related behavior* (18), safety protocol are necessary when employing EMA with individuals experiencing suicidal ideation. Therefore, we implemented some actions for ethical considerations, including a safety plan at baseline and a timely response to participants reporting high risk. A recent study found decreases in suicidal thoughts and negative feelings after intervening on high-risk responses were being triggered, but the effect on future intent ratings did not reach statistical significance. Additionally, the study found that adolescents were more inclined than adults to reduce their suicide intent ratings after triggering the warning threshold (25). Thus, these measures may have influenced the observed results.

This study had some limitations. Firstly, recent studies have utilized a higher frequency of assessments (i.e., 6–10 times/day) compared to our study, which assessed suicidal thoughts three times a day. Secondly, similar to previous EMA studies (26–29), we modified the ninth item of the PHQ-9 to evaluate suicidal ideation. However, this method has limitations as previous research used a single item to assess suicidal ideation (26–29). It cannot differentiate between active and passive suicidal ideation (27), potentially resulting in a lower rate of detection for suicidal ideation (30). Thirdly, the effect size in this study is relatively small, consistent with previous findings (9). It suggested that the importance of larger sample sizes in future studies to enhance our conclusions. Additionally, it might be necessary to account for confounding variables that could potentially impact the outcomes of this study, such as the effect of safety protocol, to ascertain true effects.

In conclusion, we found no evidence to support the notion that repeated assessment of suicidal thoughts is iatrogenic. Future research should continue to investigate the impact of frequent assessment on suicidal thoughts, taking into account individual differences, intervening on high-risk responses, and employing larger sample sizes with higher assessment frequency. This would provide a more comprehensive understanding of the potential effect associated with frequently assessing suicidal thoughts in real-time monitoring studies.

## Data availability statement

The data and relative materials supporting the conclusions of this article will be made available by the corresponding author, upon reasonable request.

## Ethics statement

The studies involving humans were approved by Ethical review boards of the Xiangya School of Public Health, Central South University (XYGW-2021-73 and XYGW-2022-39), the Affiliated Brain Hospital of Guangzhou Medical University (2021–089) and Tianjin Anding Hospital (2023–01 and 2023–02). The studies were conducted in accordance with the local legislation and institutional requirements. Written informed consent for participation in this study was provided by the participants’ legal guardians/next of kin.

## Author contributions

TC: Writing – original draft, Software, Methodology, Formal analysis. LN: Writing – review & editing, Supervision, Methodology, Funding acquisition, Conceptualization. JZ: Writing – review & editing, Investigation, Conceptualization. XH: Writing – review & editing, Resources. HT: Writing – review & editing, Resources. YM: Writing – review & editing, Resources. VS: Writing – review & editing, Conceptualization. KL: Writing – review & editing, Resources, Conceptualization. LZ: Writing – review & editing, Resources, Conceptualization.

## Footnotes

^1^Zhu J, Niu L, Hou X, Tao H, Ma Y, Silenzio V, et al. Feasibility and acceptability of ecological momentary assessment to assess suicide risk among young people with mood disorder: A mixed-methods study in Chinese culture. (2023).
