# Supplementary material for: Effects of frequent assessments on the severity of suicidal thoughts: an ecological momentary assessment study
Source: Front Public Health. 2024 May 17;12:1358604. doi: 10.3389/fpubh.2024.1358604 (PMC11141048; doi:10.3389/fpubh.2024.1358604)
Supplement: Supplementary file 1 [file Data_Sheet_1.docx]

Supplementary appendix

Contents

**The online EMA platform**…………………………………………………………2

**Introduction of Statistical method**………………………………………………..3

**MCMC diagnosis and Posterior predictive check**……………………………….5

**Sensitive analysis**…………………………………………………………………..6

**Figure S1 The interface of Xunkang assessment system**………………………..7

**Figure S2 The mini program code of Xunkang Assessment in Wechat**………..8

**Figure S3 Posterior predictive check in both models**……………………………9

**Table S1** **The information regarding the participants who withdrew from the study**………….…………………………………………………………………….10

**Table S2 Rhat and ESS of MCMC diagnosis in two-level and three-level model**………………………………………………………………………………11

**Table S3 The results of the first 14 days and second 14 days based on the original models**..…………….………………………………………………………………12

**The online EMA platform**

Participants filled in the online questionnaire based on the Wechat mini program Xunkang assessment system, the detail interface, and the link see **Figure S1** and **Figure S2**.

**Introduction of Statistical method**

As suicidal thought was an ordinal and categorical variable, so we used the cumulative ratio distributional family and a logit link function. Besides normal distribution prior (0,100) for all regression coefficients, we used the default prior of brms package for intercepts, standard deviations, and random effects correlation matrix.

Cumulative ratio model

The cumulative ratio model is a common model for analyzing ordinal variables as dependent variables. For ordinal variables with n classes, as response variables, x_1_, x_2_, x_3_... x_q_ is the independent variable, and the following formula can be established:

$$logit\left[ p\left( Y\leq j | X \right) \right]=ln\frac{p\left( Y\leq j | X \right)}{1-p\left( Y\leq j | X \right)}=\alpha_{i}+\sum_{i=1}^{q} \beta_{i}x_{i},(j=1，2，3，\ldots\ldots，n)$$

n categorical rank variables can be used to establish n-1 regression equations with n-1 intercepts. In this study, the independent variable is seven categories with 6 intercepts.

Two-level model

In light of the nested structure of our data, we employed both two-level and three-level models. In the two-level model, each instance of suicidal ideation measurement is nested within an individual. Here, 'i' means the number of measurements, and 'j' means to different participants.

Level 1: $logit\left[ p\left( Y_{ij}\leq q | X \right) \right]=\beta_{0j}+\beta_{1j} number of surveys+\beta_{2j}positive emotion+ \beta_{3j}negative emotion+ \beta_{4j}suicidal ideation in baseline+ e_{ij} （q=1，2，3，\ldots\ldots，6）$

Level2：$\beta_{0j}=\gamma_{00}+\mu_{0j}$

$\beta_{1j}=\gamma_{10}+\mu_{1j}$

*β_0j_* represents the initial level of suicidal thoughts of an individual k, and β*_1j_* represents the average rate of change of suicidal thoughts of an individual j with the increasing number of surveys. γ*_00_* represents the average initial condition of suicidal ideation for all individuals, and γ*_10_* represents the average rate of change of suicidal ideation for all individuals as the number of surveys increases.

Three-level model

In the three-level model, each instance of suicidal ideation measurement is nested within a day, which in turn is nested within an individual. In this model, 'i' means each measurement, 'k' means the day, and 'j' means different participants. The following equation can be established for the three-level cumulative ratio model in this study:

Level1：$logit\left[ p\left( y_{ikj}\leq q | X \right) \right] =\beta_{0kj}+\beta_{1kj}number of surveys+\beta_{2kj}positive emotion+\beta_{3kj}negative emotion+\beta_{4kj}suicidal ideation in baseline（q=1，2，3，\ldots\ldots，6）$

Level2：$\beta_{0kj}=\gamma_{00j}+\mu_{0kj}$

$\beta_{1kj}=\gamma_{10j}+\mu_{1kj}$

Level3：$\gamma_{00j}=\pi_{000}+r_{00j}$

$\gamma_{10j}=\pi_{100}+r_{10j}$

β_0kj_ represents the initial level of k's suicidal ideation on a certain day, and β_1kj_ represents the average rate of change of k's suicidal ideation with increasing surveys on a certain day. γ_00j_ represents the initial condition of individual j's suicidal thoughts, and π_000_ is the total average of suicidal thoughts in the initial condition. γ_10j_ is the average daily rate of change in suicidal thoughts of individual j, and π_100_ is the total average daily rate of change in suicidal thought.

**MCMC diagnosis and Posterior predictive check**

The two-level model was set as 4 chains with 2000 iterations, and the burn-in period was set as 1000 iterations, which produced 4000 posterior sample data. The three-level model is set to 4 chains and 5000 iterations, and the burn-in period is set to 2500 iterations, resulting in 10000 posterior sample data. Then, we should ensure that the MCMC converges so that the posterior distribution can be estimated accurately. It indicated that chains of MCMC converge if the extent of overlap of chains is high by observing the track diagram. Besides, Rhat and effective sample size (ESS) should be examined, which were associated with the convergence and resolution of chains. posterior predictive check is an important part of the modeling process, which helps us evaluate whether the fitted model is reasonable and accurate given the known posterior predictive distribution and actual data**.** The effective sample size (ESS) of the two-level models ranged from 1533 to 6226, and the Rhat were all less than 1.01. In the three-level model, the effective sample size (ESS) was between 880 and 9143, and the Rhat were also all less than 1.01, indicating the convergence of MCMC (detail see **Table S2**). We can derive from **Figure S3** that the posterior predictive distribution nearly recovered original data distribution.

**Sensitive analysis**

In addition, we divided the 28-day survey into two equal intervals of 14 days each and reanalyzed the data (details see **Table S3).**


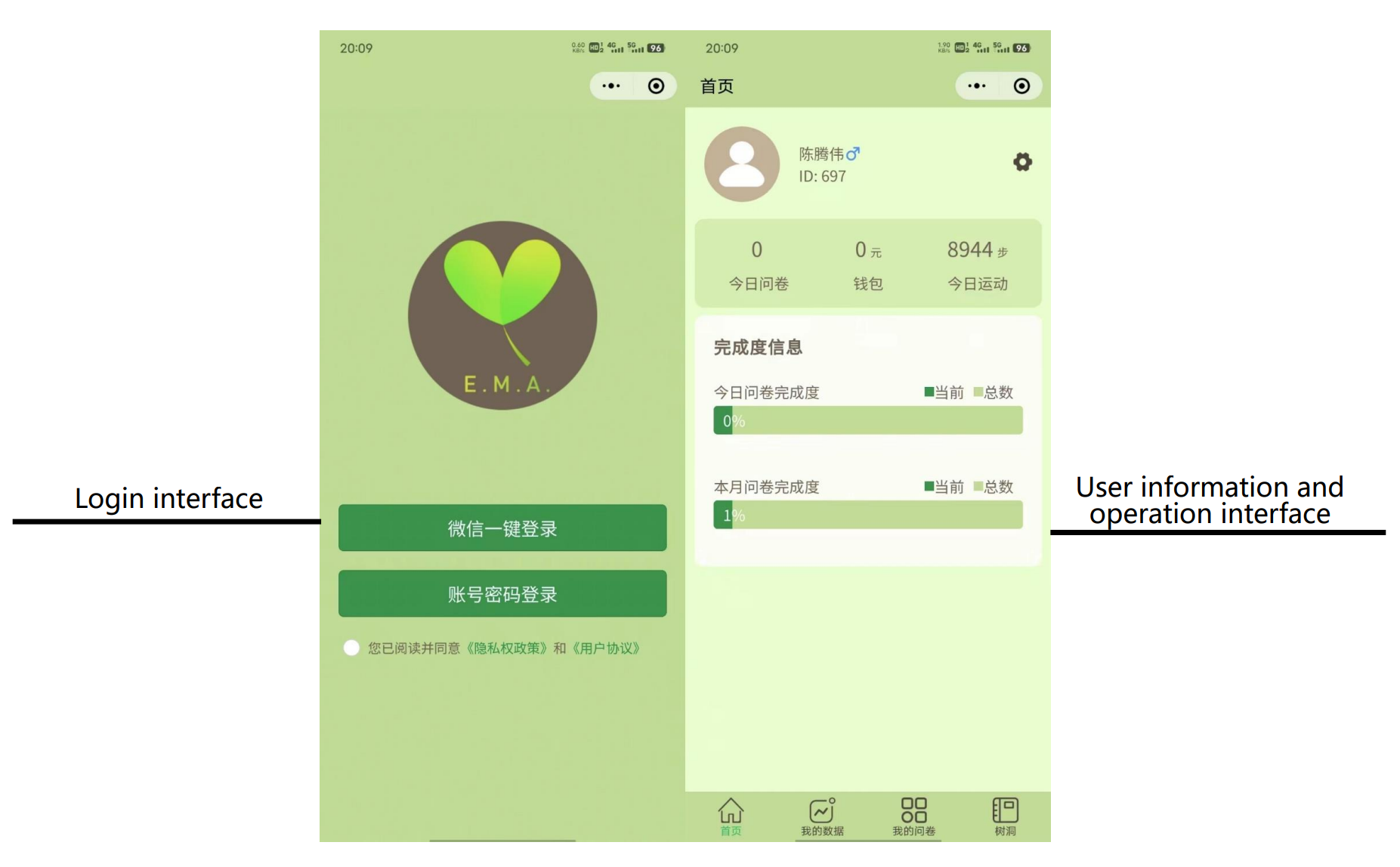


**Figure S1 The interface of Xunkang assessment system**


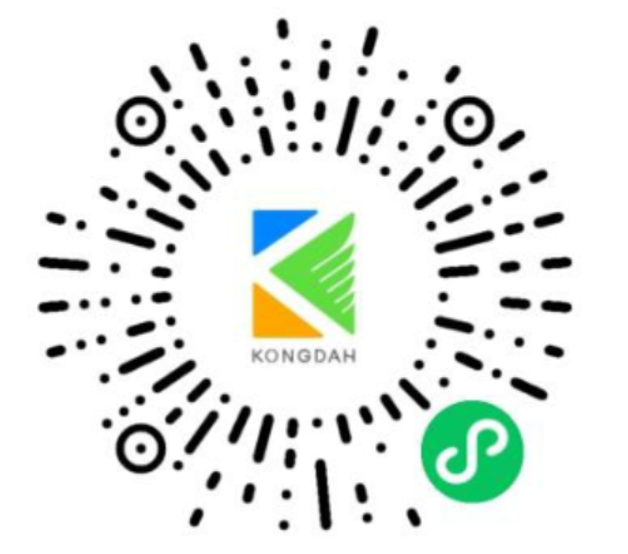


**Figure S2 The mini program code of Xunkang Assessment in Wechat**

**Figure S3 Posterior predictive check for (A) two-levels Bayesian cumulative ratio model and (B) three-levels Bayesian cumulative ratio model.** y_rep_ stands for 100 repeated samples from the posterior predictive distribution. y represents actual data. These two figures showed that the model fitted well with original data.


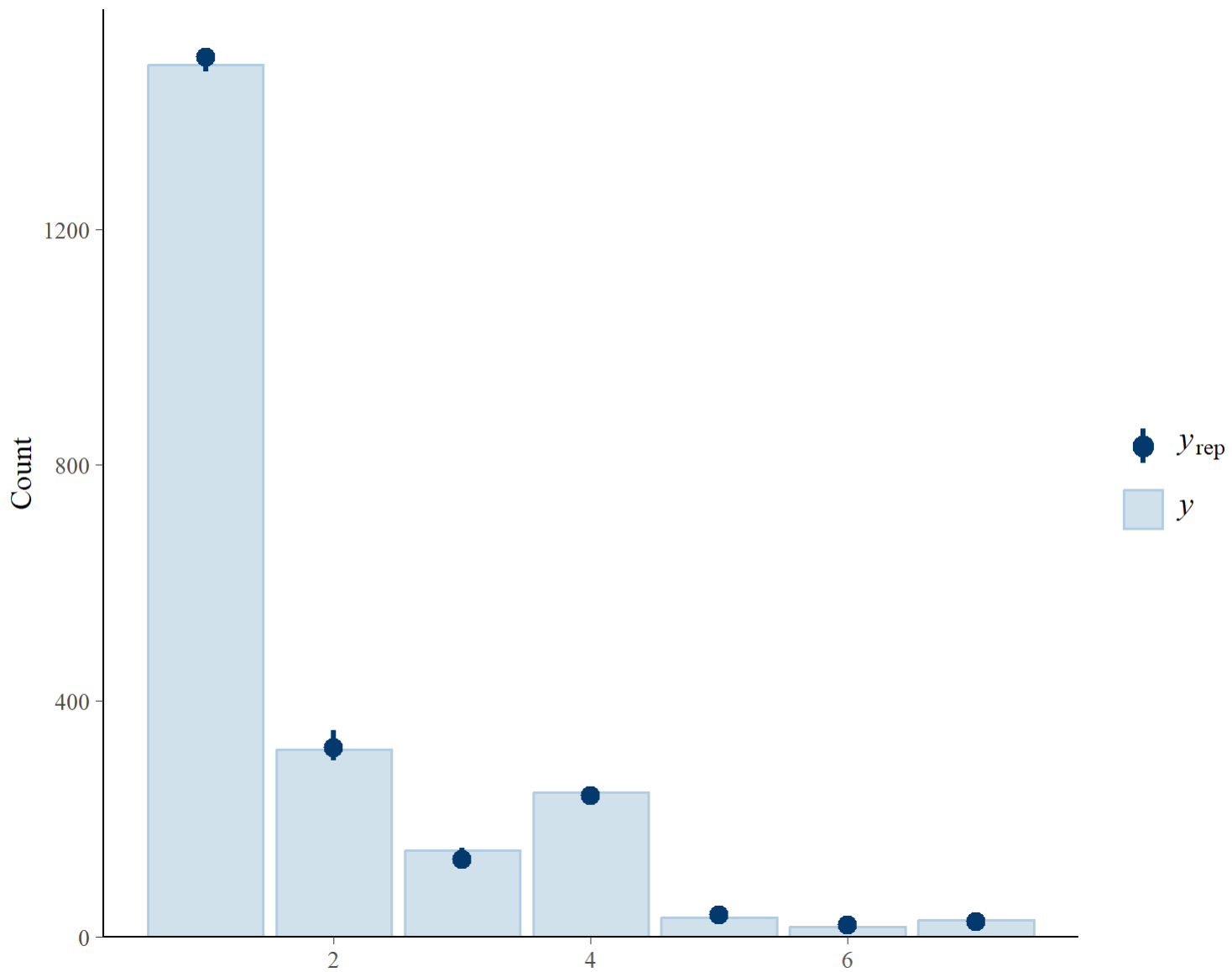


**(B)**


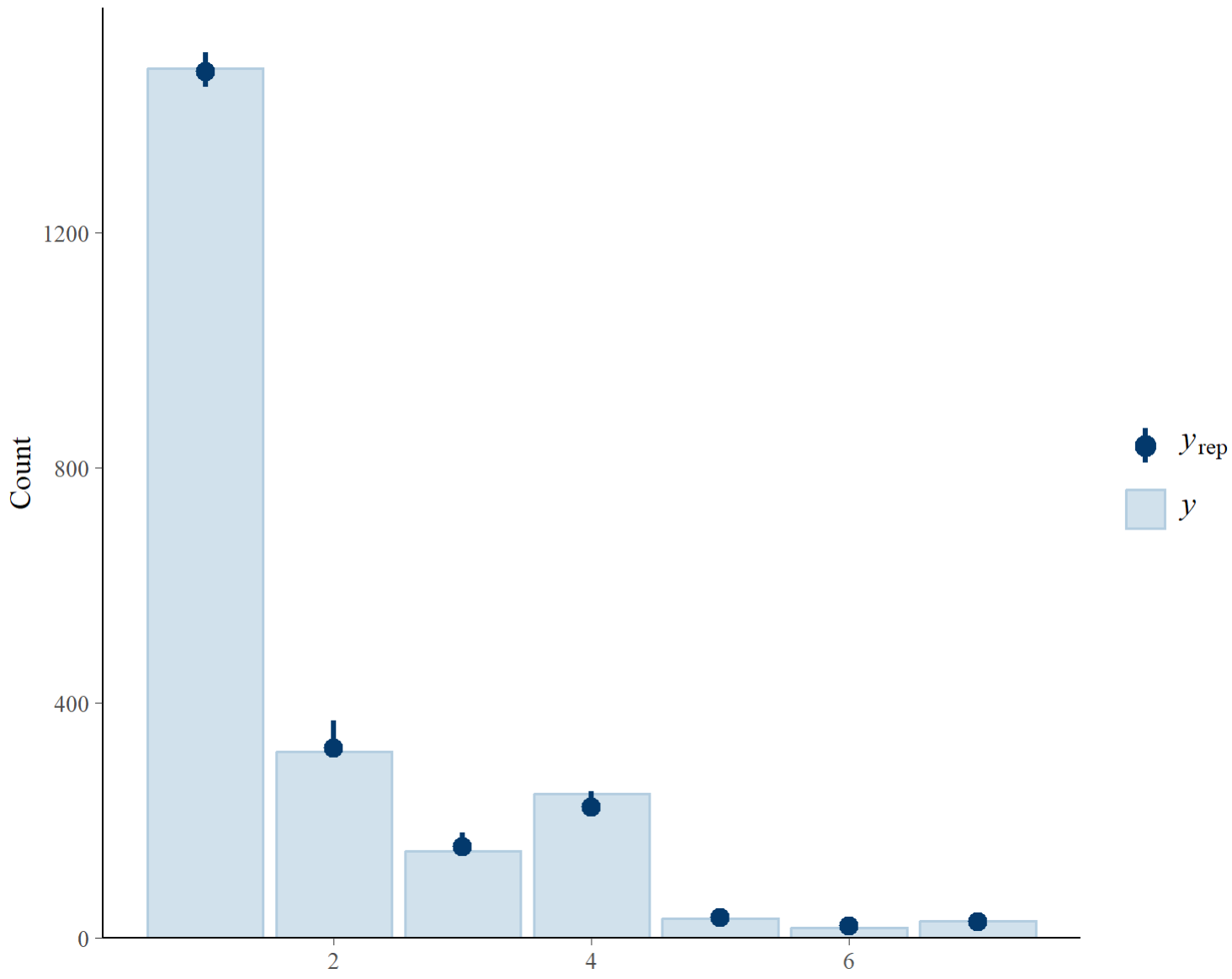


**Table S1**. The information regarding the participants who withdrew from the study.

| Sex | Age | Response days | Reasons for dropout |
| --- | --- | --- | --- |
| Female | 31 | 16 | Fear of being observed while answering questions. |
| Female | 15 | 22 | frequently forgetting the surveys and feeling bored |
| Female | 23 | 9 | hospitalized |
| Female | 23 | 21 | did not have sufficient leisure time |
| Male | 20 | 11 | hospitalized |
| Female | 19 | 14 | unwilling to continuously join in it |
| Male | 31 | 4 | feeling burdensome |
| Female | 16 | 7 | unable to respond due to mobile phone restrictions at school |
| Female | 26 | 4 | hospitalized |
| Female | 22 | 7 | Frequent answering of questions made him feel agitated |
| Female | 19 | 21 | did not have sufficient leisure time |
| Female | 14 | 25 | hospitalized |
| Male | 15 | 7 | unable to respond due to mobile phone restrictions at school |
| Female | 16 | 3 | feeling bored |
| Female | 16 | 8 | unable to respond due to mobile phone restrictions at school |
| Male | 27 | 5 | feeling psychological stress |
| Male | 17 | 7 | unable to respond due to mobile phone restrictions at school |

**Table S2 Rhat and ESS of MCMC diagnosis in two-level and three-level model.**

|  | Two-level model | | Three-level model | |
| --- | --- | --- | --- | --- |
| variable | Rhat | ESS | Rhat | ESS |
| Fixed effects |  |  |  |  |
| (Intercept 1) | 1.002 | 1570 | 1.003 | 1953 |
| (Intercept 2) | 1.002 | 1686 | 1.003 | 1920 |
| (Intercept 3) | 1.003 | 1746 | 1.003 | 1892 |
| (Intercept 4) | 1.002 | 1996 | 1.003 | 1858 |
| (Intercept 5) | 1.002 | 2134 | 1.002 | 1885 |
| (Intercept 6) | 1.001 | 2342 | 1.002 | 1910 |
| Suicidal thought at t-1 | 0.999 | 6287 | 1.001 | 3019 |
| Number of surveys | 1.001 | 2347 | 1.000 | 4316 |
| Positive emotion | 1.000 | 5474 | 1.000 | 9143 |
| Negative emotion | 0.999 | 6226 | 1.001 | 1912 |
| Random effects |  |  |  |  |
| level-2 |  |  |  |  |
| σ_intercept_ | 1.001 | 1710 | 1.000 | 880 |
| σ _observation number_ | 1.002 | 1533 | 1.003 | 944 |
| level-3 |  |  |  |  |
| σ_intercept_ |  |  | 1.002 | 2681 |
| σ _observation number_ |  |  | 1.003 | 1319 |

Note: ^a^ Rhat: It also was called potential scale reduction factor (PSRF), which measurs the convergence of MCMC.

^b^ ESS: Effective Sample Size measures the stability of MCMC.

|  | Within the first 14 days | | | | Within the second 14 days | | | |
| --- | --- | --- | --- | --- | --- | --- | --- | --- |
| variable^a^ | Two-level model | | Three-level model | | Two-level model | | Three-level model | |
|  | Median | 95%HDI | Median | 95%HDI | Median | 95%HDI | Median | 95%HDI |
| Fixed effects |  |  |  |  |  |  |  |  |
| Number of surveys | -0.04 | -0.08,-0.02 | -0.17 | -0.44, 0.06 | -0.03 | -0.08, 0.02 | -0.21 | -0.67, 0.16 |
| Random effects |  |  |  |  |  |  |  |  |
| level-2 |  |  |  |  |  |  |  |  |
| σ _observation number_ | 0.08 | 0.05,0.11 | 0.17 | 0,0.41 | 0.07 | 0.01,0.16 | 0.12 | 0.00, 0.42 |
| level-3 |  |  |  |  |  |  |  |  |
| σ _observation number_ |  |  | 0.22 | 0,0.48 |  |  | 0.24 | 0.00, 0.80 |
| Loo-R^b^ | 0.722 | | 0.711 | | 0.771 | | 0.770 | |
| Looic^c^ | 1952.3 | | 2019.0 | | 700.2 | | 711.1 | |
| Total survey number | 1621 | | | | 645 | | | |
| Number of respondents | 83 | | | | 33 | | | |

**Table S3** **The results of the first 14 days and second 14 days based on the original models.**

Note: ^a^ This table only showed the results that we are interested in the association between suicidal ideation and the number of surveys in the fixed effects and the standard deviation of this association in the random effects.

^b^ Loo-R^2^ is the R^2^ adjusted by Leave-One-Out Cross-Validation (LOO-CV).

^c^ Looic transforms the Bayesian LOO estimate of expected log pointwise predictive density into an information deviation scale
